# FACTOR STRUCTURES OF THE KOREAN VERSION OF NEED FOR COGNITION SCALE SHORT FORM (K-NFC-S) AMONG KOREAN OLDER ADULTS

**DOI:** 10.1093/geroni/igad104.2623

**Published:** 2023-12-21

**Authors:** Juhyeong Lee, Gaeun Han, Yeonsoo Shin, Giyeon Kim

**Affiliations:** Chung-Ang University, Seoul, Republic of Korea; Chung-Ang University, Seoul, Republic of Korea; Chung-Ang University, Seoul, Republic of Korea; Chung-Ang University, Seoul, Republic of Korea

## Abstract

This study aimed to examine the underlying factor structures of the Korean Version of Need for Cognition Scale Short Form (K-NfC-S) among older adults in South Korea. A nationally representative sample of Korean older adults aged 65 or older (N=2,281) was drawn from the 2020 Korean Media Panel Survey. After dividing the sample into two groups at random, both exploratory (N=1,117) and confirmatory (N=1,164) factor analyses were conducted respectively using SPSS 26.0 and AMOS 26. A model fit was assessed with the Tucker-Lewis Index (TLI), the comparative fit index (CFI), and the root-mean-square error of approximation (RMSEA). Results from the exploratory factor analysis revealed that K-NfC-S were divided into two domains. Factor I consisted of eleven positively worded items and Factor II consisted of four negatively worded items. However, in the confirmatory factor analysis, a single-factor model with correlated errors among the negatively worded items showed the best fit among the postulated models of K-NfC-S (TLI=.917, CFI=.934, RMSEA=.074). Consistent with previous findings, the results from the present study support a single dominant factor structure underlying responses to the K-NfC-S items among older adults in South Korea. The findings suggest that the two-factor structure identified from exploratory factor analysis may be an artifact of method effects associated with the negatively worded items included in the K-NfC-S.

